# Predictors of breast self - examination among female teachers in Ethiopia using health belief model

**DOI:** 10.1186/s13690-015-0087-7

**Published:** 2015-08-27

**Authors:** Negussie Birhane, Abebe Mamo, Eshetu Girma, Shifera Asfaw

**Affiliations:** SNNP Health Bureau, Kafa zone health office, Kafa, Ethiopia; Departments of Health Education and Behavioral Sciences, Jimma University, Jimma, Ethiopia; Department of public health, College Of Medicine, Addis Ababa University, Addis Ababa, Ethiopia

**Keywords:** Breast self examination, Breast cancer, Female, Teachers and Health Belief Model

## Abstract

**Background:**

Breast cancer is by far the most frequent cancer of women. It is the second leading cause of death in women worldwide. Approximately one out of eight women develops breast cancer all over the world. Majority of cases of cancer of the breast are detected by women themselves, stressing the importance of breast self-examination. The main objective of this study was to assess predictors of breast self-examination among female teachers in Kafa Zone, South West part of Ethiopia.

**Methods:**

A cross-sectional study was conducted among randomly selected 315 female teachers. Self administered a structured questionnaire including socio-demographic characteristics, knowledge about breast cancer and perception of teachers on breast self examination using the Champion’s revised Health Belief Model sub scales used as data collection instrument. Multivariable logistic regression analyses were used to identify independent predictors of breast self -examination performance.

**Result:**

Three hundred and fifteen female teachers were participated in this study. Their mean age was 33 SD [±7] years. Only 52 (16.5 %) participants ever heard about breast self examination and from those who heard about breast self examination 38 (73.07 %) of them ever performed breast self examination. After controlling for possible confounding factors, the result showed that knowledge towards breast self examination, perceived susceptibility, perceived severity and the net perceived benefit were found to be the major predictors of breast self examination.

**Conclusion:**

This study revealed that breast self examination performance among female teachers was very low. Therefore, behavior change communication and interventions that emphasize different domains that increase the perceived threat to breast cancer as well as on the benefits of breast self-examination to increase the perception of the teachers in an integrated manner may be the most effective strategies that should be considered by the health offices and educational offices. These may help to increase the knowledge and skill of female teachers on how to perform breast self-examination and its importance hence helpful for wider of the community.

## Background

Globally, breast cancer is the most common cancer among women, comprising 23 % of the female cancers. It is also the primary cause of cancer-related deaths in low-income countries [1, 2]. One in eight women born today will be diagnosed with breast cancer at some time in her life [3].

Early detection of breast cancer plays an important role in decreasing its morbidity and mortality. Breast self-examination (BSE) is one of the screening methods for early detection of breast cancer. However, in developing countries women do not perform breast self-examination for various reasons [4].

Over the past two decades, breast cancer has become a matter of serious public health concern in developing countries due to a high incidence of this cancer and associated mortality, especially among women [5]. The burden of cancer in developing countries is increasing because of the aging and growth of the population as well as increased prevalence of risk factors associated with economic transition, including smoking, obesity, physical inactivity, and reproductive behaviors [6].

BSE is a sort of examination made by each woman subsequent to the age of 20 and it is an economical, easy-to-apply, safe, non-invasive procedure with no special material or tool requirements; and it is an effective screening method for breast cancer which only takes five minutes to apply [7].

Women who carry out BSE on a regular basis have been a possibility of easily recognizing both the appearance and feel of their breasts hence often helps them to detect any changes early. However, if improperly done, BSE has the risk of giving false health security and may actually reduce willingness to undergo screening [8].

A study conducted in a rural area of western Turkey shows that the women’s responses 23.4 % of them had no knowledge about breast cancer, 27.9 % had no concept of BSE, 89.3 % had never had a mammography and 75.0 % had never had breast cancer examination [9]. Study in exploration of barriers to breast self examination carried out in Malaysia showed that 77.5 % believed that BSE is important for early detection of breast cancer and 55 % of respondents have the experience of performing BSE; among those who practice BSE, only 2 8.5 % of them practice BSE once a month [10].

The other study on female secondary school teachers in Ilorin, Nigeria showed that most of the respondents 95.6 % had heard about BSE at one time or the other. Majority of the respondents, 54.8 % of them had done BSE before while 5.2 % had never done it. One hundred and sixty 49.0 % of the respondents who knew about the procedure were currently doing it while 50.9 % were not [11].

Early detection or screening is the most effective method to reduce morbidity and mortality from breast cancer. Certain methods like clinical breast examination, BSE and mammography have been defined as activities facilitating the early screening and improving health of females and good for early detection of breast cancer [12].

Mammography is the method of choice for the early detection of breast cancer. However its limited use in developing countries due to the high cost and limited availability make BSE a convenient and cost effective method, while less reliable [13–15].

Communicable and chronic diseases are the major health issues in Ethiopia and all the efforts and recourses are allocated into it. Government, non government organizations and international partners all are giving their at most effort to deal with these diseases. For a long period cancer, predominantly breast cancer is on the bottom of their priority list [2] and a study in Ethiopian Tikur Anbesa Radiotherapy center showed that cancer was reported the second out of the ten top cancers registered [4].

It is easily understandable that the incidence and mortality of breast cancer is growing at a fast rate. But as we do not have any cancer registry along with relevant data were not well documented it is difficult to say the exact circumstances in Ethiopia. But as one study on health extension workers [HEW] stated only 14.4 % practiced BSE regularly (every month) and 147 (37.3 %) HEWs reported that they practiced BSE during their life time [4].

In most Ethiopia region and zones access to health care services, especially comprehensive diagnostic services is very low, in some areas completely unavailable hence, individual self health empowerment is very important. Teachers are the best examples in educating and implementing their activities throughout the country, from the big cities to very remote rural villages in Ethiopia. For such reason, female teachers are not only educators, but serve as role models and change agents who often offer useful counsel on health promotion especially for the students and the community.

Therefore, Studies have not been conducted so far on the perception of BSE among female teachers in Ethiopia. Hence, findings from this study will provide a starting point for female teachers to raise awareness amongst themselves, in the community as well as among their female students about breast cancer and the role of BSE. Moreover, findings from the study can provide information on BSE for governmental health officials and other nongovernmental organizations which are working on cancer particularly breast cancer to raise awareness amongst women and the role of BSE in breast cancer prevention and control program.

## Methods

### Study area and period

A cross sectional study was conducted from March 1 to March 30, 2013 among 316 randomly selected female teachers. The study was conducted in Kaffa zone; southern part of Ethiopia located 458 Km south west of Addis Ababa. Kaffa zone is administratively divided in to 10 Woreda (districts), 312 kebele (the lowest administrative unit) (296 rural and 16 urban) with a projected total population of 1. 1million; in which 50.8 % of them were female and 49.2 % of them were male. The zone has one hospital, 36 health centers, 264 health posts and 32 private clinics with potential basic health service coverage of 94 % which was reported by the zonal officials. In the academic year 2013 there were 3209 male and 1175 female teachers were available from primary to college levels [16].

### Study population

The study population was randomly selected female teachers. The sample size was determined using single population proportion formula at 95 % of confidence interval with assumption of prevalence of BSE practice were 54.8 % [11] with 5 % precision and 10 % was added for possible non-response and the final sample size was 316 female teachers.

### Sampling technique

To collect the data, initially all schools in Kaffa zone were identified and listed. From all listed schools female teachers were identified then from each school all teachers with the age of 20 and above were selected and listed for the study based on the proportion of the number of teachers in each school using simple random sampling technique.

### Measurements

A self administered questionnaire including socio-demographic characteristics, knowledge and Health Belief Model constructs were used as data collection instruments. Most items offered five response choices ranging from “strongly disagree (scores 1 point)” to “strongly agree (scores 5 points)”. Susceptibility of breast cancer consisted of three items scored from 3 to 15, seriousness of breast cancer consisted of six items scored from 6 to 30, BSE benefits consisted of four items scored from 4 to 20, BSE barriers consisted of eight items scored from 8 to 40, BSE self-efficacy consisted of 10 items scored from 10 to 50 and cues to action consisted of 6 items with ‘yes or no’ questions. For all constructs of Health Belief Model the higher scores indicated having high perception towards performing BSE except for barriers to BSE in which higher score indicated high barrier to perform BSE. For perceived net benefit we used the sum score of perceived benefit minus perceived barriers. Knowledge about BSE was also assessed with 5 items and the responses of all items were sum up and higher scores indicated having high knowledge towards performing BSE. The reliability coefficient for each subscale was calculated using Cronbach’s alpha. Cronbach’s alpha coefficients for the original Health Belief Model constructs for susceptibility, seriousness, BSE benefits, BSE barriers and BSE self-efficacy were 0.87, 0.80, 0.69, 0.83 and 0.90 respectively [17]. The reliability coefficient for the constructs of Health Belief Model for this study were 0.78, 0.76, 0.64, 0.81 and 0.82 for susceptibility, seriousness, benefits, barriers and self-efficacy to BSE respectively.

Practice of breast self examination (BSE) was assessed using item with the responses of “Yes or No” type like “have you ever perform BSE for screening of cancer?” Those who responded “Yes” considered as they were practicing breast self examination.

Data was supervised by teachers who were trained on the objective of the study, method of data collection and content of questionnaire to avoid any ambiguity raised during data collection. Data was checked for completeness, accuracy, and consistency by supervisors and principal investigator after the data collection on daily base. Questionnaire was prepared first in English and translated into Amharic and retranslated back to English to check for consistency. The prepared questionnaire was pre-tested on 5 % of female teachers those who were not be included in the study to identify the clarity and sequence of question.

Data were coded and entered to computer using Epi data version 3.1 and exported to SPSS program version 16.0 for further analysis. The result was presented using frequency tables and percentage. Bivariate analysis was done to determine association between factors and breast self examination. Multivariate logistic regressions was performed to identify the independent predictors of breast self examination.

Ethical approval was obtained from the Jimma University Ethical Review Board. Letter of cooperation to each district were obtained from Kaffa Zone Education Department. Written informed consent was also obtained from each study participants.

## Results

### Socio-demographic characteristics

A total of 315 study subjects participated in this study making response rate of 99.6 %. The mean age of the study population was 33 (SD ± 7) years. Concerning marital status 252 (80 %) of the participants were married. Majority 254 (80.5 %) were Kaffa by ethnicity and 260 (82.5 %) were Orthodox religion followers. The mean monthly income of the study population was 2034 (SD ± 532) (1USD ≈ 20ETHBR) Ethiopian Birr (Table 1).

**Table 1:** Socio-Demographic characteristics of female teachers in Kafa zone south west Ethiopia March, 2013 (N = 3 15)

Category	Frequency	Percent
Marital status		
Single	37	11.7
Married	252	80.0
Divorced	26	8.3
Education		
Certificate	113	35.9
Diploma	166	52.7
Degree	36	11.4
Ethnicity		
Kafa	254	80.5
Amhara	38	12.1
Oromo	14	4.4
^a^Other	9	2.9
Religion		
Orthodox	260	82.5
Catholic	25	7.9
Muslim	23	7.4
Protestant	7	2.2

^a^Others Dawro, Gurage, Tigre and Bench

### Source of information for BSE

Majority of study participants 278 (88.3 %) ever heard about breast cancer and 52 (16.5 %) of the participants ever heard about BSE. The main source of information on breast cancer and BSE were television or radio 227 (72.06 %) and about 37 (11.7 %) of the respondents have never obtained information about either breast cancer or BSE.

### Knowledge and practices of breast self-examination

In this study 52 (16.5 %) of women heard about BSE and from those who heard about BSE 38 (73.07 %) of them screened for breast cancer. Knowledge was assessed as continuous variable with possible values ranging from 10 to 90 with the mean score of 40.14 (SD ± 24). When we see the history of breast cancer, 272 (86.3 %) of the respondents reported that they did not have any previous history of breast disease. From the total study participants 9 (2.9 %) of them reported that they had had family history of breast cancer (Table 2).

**Table 2:** Practice on Breast self-examination among female teachers of Kafa Zone south west Ethiopia March, 2013

Characteristics	Category	Frequency	Percent
Heard about breast cancer	Yes	278	88.3
	No	37	11.7
Heard about BSE	Yes	52	16.5
	No	263	81.3
Practice of BSE	Yes	38	12.1
	No	277	87.9
Family history of breast cancer	Yes	9	2.9
	No	306	97.1
History of breast disease	Yes	43	13.7
	No	272	86.3

### Perception on BSE

Perception of participants measured using Health Belief Model constructs and treated as continuous variables except for cues to action. The other five constructs (susceptibility, severity, barriers, benefits and self-efficacy) were analyzed as a continuous variable with possible values ranging from 3 to 15 for susceptibility with the mean score of 8.45 (±2.2), from 6 to 30 for severity with the mean score of 17.9 1 (±4.0), from 8 to 40 for barriers with the mean score of 16.99 (±5.4), from 4 to 20 for benefits with the mean score of 14.65 (±3.9) and from 10 to 50 for self-efficacy with the mean score of 27.74 (±9.9).

For cues to action 52 (16.5 %) of respondents had information from various sources and 263 (84.5 %) said they didn’t receive any information from any source about BSE.

### Independent predictors of BSE among Female teachers

Among socio demographic variables: Knowledge about BSE was significant in explaining BSE performance. This study showed that as participant knowledge increases the odds of performing breast self examination also increased by 1.1 times, keeping all other factors constant [AOR 1.10 (95 %, CI 1.05, 1.10)].

The binary logistic regression result showed that all constructs of health belief model were significantly associated with BSE with 95 % CI at *P*-value 0.05. But after controlling for possible confounding factors, the result showed that per a unit increases in total score of perceived susceptibility and severity towards breast cancer the odds of performing BSE increased by 1.95, [AOR 95 %,CI 1.95 (1.44–2.63)] and 1.24 [AOR, 95 %,CI 1.24 (1.11–1.46)] respectively.

The other variable which independently associated with BSE is perceived net benefits, which is the sum score of perceived benefit minus perceived barriers; in which a unit increase in total score of perceived net benefits the likely hood of performing breast self examination was increased by 1.10 [AOR, 95 % CI, 1.10 (1.03–1.20)] (Table 3).

**Table 3:** Multiple logistic regression analysis of variables and BSE among female teachers in Kaffa zone, May, 2013

Variables	BSE Practice		COR (95 % CI)	AOR (95 % CI)
Category	No	Yes		
Marital status				
Single	32	5	. 66 (.17–2.25)	
Married	224	28	.53 (.18–1.50)	
Divorced	21	5	1	
Educational status				
Certificate	107	6	.19 (.06–.61)	.24 (.05–1.06)
Diploma	142	24	.59 (.24–1.45)	.70 (.19–2.5)
Degree	28	8	1	1
Ethnicity				
Kafa	228	26	.93 (.11–7.58)	1
Amhara	29	9	2.48 (.27–22.6)	1.94 (.74–5.1 1)
Oromo	12	2	1.33 (.10–17.3)	.88 (.16–4.88)
^a^Other	8	1	1	.89 (.09–8.30)
Religion				
Orthodox	225	35	3.72 (.48–28.4)	
Catholic	24	1	1.2 (.06–18.5)	
Muslim	22	1	4.00 (.22–73.6)	
Protestant	6	1	1	
Family history of breast cancer				
Yes	9	0	1	
No	268	38	0.0 (0.0–0.0)	
History of breast disease				
Yes	33	10	1	1
No	244	28	.38 (.17–.85)	3.44 (.87–13.57)
Age^b^			1.051 (1.01–1.17)	1 .06 (.99–1.13)
Knowledge^b^			1.1 (1.04–1.07)	1.1 (1.05–1.10)^a^
Monthly income^b^			1.00 (.99–1.01	
Susceptibility^b^			1.58 (1.31–1.90)	1.95 (1.44–2.63)^a^
Severity^b^			1.11 (1.02–1.22)	1.24 (1.11–1.46)^a^
Net Benefit^b^			1.12 (1.06–1.2)	1.10 (1.03–1.20)^a^
Self efficacy^b^			1.10 (1.03–1.11)	1.04 (.98–1.10)

^a^Variables with *P*-value < 0.05; ^b^Continuous Variables

## Discussion

This study revealed that very few teachers, 38 (12 %) performed breast self-examination among sampled school female teachers. This is inconsistent with study conducted in female secondary school teachers in Ilorin, Nigeria and rural community in Oyo State, [11, 18]. This might be due to lack of community base awareness and screening program of breast self examination in our country more of in study area. The other possible explanation about this difference may be due to difference in educational status, accessibility to information or mass media and composition of the study population.

On the other hand this study is consistent with the study conducted on health extension workers in western Ethiopia in which the study stated that about 14 % of them performed BSE [4].

This study showed that as a unit increase in total score of knowledge of female teachers’ the likely hood of performing BSE was also increased by 1.1. This finding was consistent with study conducted in Turkish academic women and housewives, and also congruent with similar study among female secondary school teachers in Ilorin, Nigeria [11, 19]. This may be explained by the fact that knowledge of breast cancer and BSE was recognized as a necessary precursor to women’s adherence performance of BSE.

From Health Belief Model constructs, perceived susceptibility, perceived severity and the net benefit to perform BSE were independently significant in explaining the performance BSE. On other hand self efficacy and cues to action towards the practice of BSE were not found to be independent predictors.

Perceived susceptibility of breast cancer has shown statistically significant association with BSE performance. Our study showed that as a unit increase in total score of perceived susceptibility the likely hood of performing breast self examination was also increased by 2. This finding is consistent with different studies conducted in Turkey [20, 21]. This might be explained as the study participants those having high susceptibility may belief that early detection has the potential to improve cancer outcomes.

Similarly as a unit increases in total score of perceived severity the odds of breast self examination performance was increased by 1.2. This finding was in line with studies conducted in Nigeria and Kuwaiti [13, 22]. This might be due to the individual beliefs about the seriousness of the disease and possible outcome of the disease. The other explanation may be high perceived susceptibility and severity towards breast cancer may also increase the perceived threat of respondents; hence the participants can perform BSE. In general, a teacher who perceived as she is highly susceptible to breast cancer and that she perceived breast cancer is a serious disease, she would be more likely to perform breast examinations.

The other predictor variable towards BSE was perceived net benefit, it was significantly associated with BSE and it indicated that as a unit increases in sum score of perceived benefits minus perceived barriers which is the net benefit; the odds of breast self examination performance also increased by 1.1. Similar finding was reported from cross-sectional study conducted in Kuwaiti female school teachers and Turkish women [21, 22]. This might be due to teachers were open to adopt health behaviors despite early diagnosis to reduce risk of getting breast cancer. The other important variable that was not independently predicts BSE was self-efficacy. This finding is inconsistent with the study in Iran on school teachers [23, 24]. The results revealed that the women with greater perceived self-efficacy were more likely to perform BSE than those with lower perceived self-efficacy. This inconsistency may be due to the difference of education level, media exposure and culture of the participants.

### Limitation of the study

Limitations of this work include that we did not collect detail data regarding mammography and the availability of other screening methods in the community. The other major limitation was lack of related papers as well as published on reputable journals in Ethiopia and developing countries; so only specific and available references were used.

## Conclusions

This study tried to assess predictors of breast self examination among female teachers and it revealed that the practice of BSE was very low. The study evidenced that, participant’s having good knowledge, the extent to which teachers perceived they are susceptible to cancer, perceived that cancer is severe, and feel benefitted from BSE has surfaced as some of the most important predictors to affect teacher’s decisions about performing BSE. Strategies that increase the awareness of females about breast cancer would be required. Health professionals and health extension workers should promote breast health education program at school and community level by focusing on the threat of cancer, and benefit of BSE since these factors found as the independent predictors of teachers’ decisions about performing BSE.

## Abbreviations

AOR: Adjusted odds ratio
BSE: Breast self-examination
CI: Confidence interval
COR: Crude odds ratio
HEW: Health extension workers
SD: Standard deviation
SNNP: Southern Nation and Nationality People
SPSS: Statistical packages for social science
WHO: World Health Organization
